# The MetaCyc database of metabolic pathways and enzymes and the BioCyc collection of pathway/genome databases

**DOI:** 10.1093/nar/gkv1164

**Published:** 2015-11-02

**Authors:** Ron Caspi, Richard Billington, Luciana Ferrer, Hartmut Foerster, Carol A. Fulcher, Ingrid M. Keseler, Anamika Kothari, Markus Krummenacker, Mario Latendresse, Lukas A. Mueller, Quang Ong, Suzanne Paley, Pallavi Subhraveti, Daniel S. Weaver, Peter D. Karp

**Affiliations:** 1 SRI International, 333 Ravenswood, Menlo Park, CA 94025, USA; 2 FCEN, University of Buenos Aires and CONICET, Argentina; 3 Boyce Thompson Institute for Plant Research, Tower Road, Ithaca, NY 14853, USA

## Abstract

The MetaCyc database (MetaCyc.org) is a freely accessible comprehensive database describing metabolic pathways and enzymes from all domains of life. The majority of MetaCyc pathways are small-molecule metabolic pathways that have been experimentally determined. MetaCyc contains more than 2400 pathways derived from >46 000 publications, and is the largest curated collection of metabolic pathways. BioCyc (BioCyc.org) is a collection of 5700 organism-specific Pathway/Genome Databases (PGDBs), each containing the full genome and predicted metabolic network of one organism, including metabolites, enzymes, reactions, metabolic pathways, predicted operons, transport systems, and pathway-hole fillers. The BioCyc website offers a variety of tools for querying and analyzing PGDBs, including Omics Viewers and tools for comparative analysis. This article provides an update of new developments in MetaCyc and BioCyc during the last two years, including addition of Gibbs free energy values for compounds and reactions; redesign of the primary gene/protein page; addition of a tool for creating diagrams containing multiple linked pathways; several new search capabilities, including searching for genes based on sequence patterns, searching for databases based on an organism's phenotypes, and a cross-organism search; and a metabolite identifier translation service.

## INTRODUCTION

MetaCyc (MetaCyc.org) is a highly curated reference database of small-molecule metabolism from all domains of life. It contains data about enzymes and metabolic pathways that have been experimentally validated and reported in the scientific literature (1). MetaCyc is a uniquely valuable resource due to its exclusively experimentally determined data, intensive curation, and tight integration of data and references. It is commonly used as a reference in various fields including biochemistry, enzymology, genome and metagenome analysis and metabolic engineering.

In addition to its role as a general reference on metabolism, MetaCyc can be used by the PathoLogic component of the Pathway Tools software (2) as a reference database to computationally predict the metabolic network of any organism that has a sequenced and annotated genome (3). During this automated process, the predicted metabolic network is captured in the form of a Pathway/Genome Database (PGDB). In addition to the automated creation of PGDBs, Pathway Tools enables scientists to improve and update these computationally generated PGDBs by manual curation. SRI has used MetaCyc to create more than 5700 PGDBs (as of September 2015), which are available through the BioCyc (BioCyc.org) website. In addition, many groups outside SRI have generated many thousands of additional PGDBs. Interested scientists may adopt any of the SRI PGDBs through the BioCyc website for further curation (biocyc.org/intro.shtml#adoption).

## METACYC ENHANCEMENTS

### Expansion of MetaCyc

Since the last *Nucleic Acids Research* publication (2 years ago) (1), we added 309 new base pathways (pathways comprised of reactions only, where no portion of the pathway is designated as a subpathway) and 10 superpathways (pathways composed of at least one base pathway plus additional reactions or pathways), and updated 171 existing pathways, for a total of 490 new and revised pathways. The total number of base pathways grew by 13%, from 2097 (version 17.5) to 2363 (version 19.1) (the total increase is less than 309 pathways because some existing pathways were deleted from the database during this period).

Further, the number of enzymes, reactions, chemical compounds, and citations in the database grew by 15%, 11%, 13%, and 23%, respectively; the number of referenced organisms increased by 9.5% (currently at 2694). See Table 1 for a list of species with >20 experimentally elucidated pathways in MetaCyc, and Table 2 for the taxonomic distribution of all MetaCyc pathways.

**Table 1.** List of species with 20 or more experimentally elucidated pathways represented in MetaCyc (meaning experimental evidence exists for the occurrence of these pathways in the organism)

Bacteria		Eukarya		Archaea	
*Escherichia coli*	329	*Arabidopsis thaliana*	335	*Methanocaldococcus jannaschii*	29
*Pseudomonas aeruginosa*	71	*Homo sapiens*	264	*Methanosarcina barkeri*	22
*Bacillus subtilis*	62	*Saccharomyces cerevisiae*	188	*Sulfolobus solfataricus*	21
*Pseudomonas putida*	51	*Rattus norvegicus*	83		
*Salmonella typhimurium*	41	*Glycine max*	62		
*Pseudomonas fluorescens*	32	*Solanum lycopersicum*	55		
*Mycobacterium tuberculosis*	31	*Pisum sativum*	55		
*Klebsiella pneumoniae*	29	*Mus musculus*	54		
*Synechocystis sp. PCC 6803*	27	*Zea mays*	48		
*Enterobacter aerogenes*	26	*Nicotiana tabacum*	46		
*Agrobacterium tumefaciens*	24	*Oryza sativa*	46		
		*Solanum tuberosum*	43		
		*Catharanthus roseus*	30		
		*Spinacia oleraca*	29		
		*Hordeum vulgare*	27		
		*Triticum aestivum*	25		
		*Bos taurus*	23		
		*Petunia x hybrida*	21		
		*Sus scrofa*	20		

The species are grouped by taxonomic domain and are ordered within each domain based on the number of pathways (number following species name) to which the given species was assigned.

**Table 2.** The distribution of pathways in MetaCyc based on the taxonomic classification of associated species

Bacteria		Eukarya		Archaea	
Proteobacteria	1115	Viridiplantae	964	Euryarchaeota	150
Firmicutes	348	Fungi	421	Crenarchaeota	41
Actinobacteria	321	Metazoa	363	Thaumarchaeota	1
Cyanobacteria	82	Euglenozoa	30		
Bacteroidetes/Chlorobi	76	Alveolata	18		
Deinococcus-Thermus	29	Amoebozoa	11		
Thermotogae	25	Stramenopiles	8		
Tenericutes	18	Haptophyceae	6		
Aquificae	17	Rhodophyta	5		
Spirochaetes	14	Fornicata	4		
Chlamydiae -Verrucomicrobia	9	Parabasalia	3		
Chloroflexi	8				
Planctomycetes	6				
Fusobacteria	4				
Nitrospirae	2				
Thermodesulfobacteria	2				
Chrysiogenetes	1				
Nitrospinae	1				

For example, the statement ‘Tenericutes 18’ means that experimental evidence exists for the occurrence of at least 18 MetaCyc pathways in members of this taxonomic group. Major taxonomic groups are grouped by domain and are ordered within each domain based on the number of pathways (number following taxon name) associated with the taxon. A pathway may be associated with multiple organisms.

### Reaction balancing

Since the last Nucleic Acids Research publication we enhanced the reaction-balance-checking algorithm, which verifies the balance of each reaction in the database, to check not only for elemental composition but also for electric charge. As of August 2015, MetaCyc contains 12 560 balanced reactions. The database also contains 1465 reactions that cannot be balanced for assorted reasons (for example, a reaction may describe a polymeric process, such as the hydrolysis of a polymer of an undefined length, may involve an ‘n’ coefficient, or may involve a substrate that does not have a defined structure, such as ‘an aldose’).

### Gibbs free energy

Standard Gibbs free energy of formation (Δf*G*′°) values have been added to most MetaCyc compounds, and standard change in Gibbs free energy (Δr*G*′°) values have been added to most MetaCyc reactions. While a small number of values were obtained from the experimental literature, most values were computed.

The computation of the standard Gibbs free energy of formation for compounds is performed in two steps using a new module of Pathway Tools. First, the Gibbs free energy is estimated for pH 0 and ionic strength of 0 (Δf*G*º), using a technique based on the decomposition of the compounds into chemical groups with known energy contributions to the larger compound (4). In the second step, the standard Gibbs free energy is calculated for pH 7.3 (the pH of an *Escherichia coli* cell, and the pH for which all compounds in MetaCyc are protonated) and ionic strength of 0.25 (Δf*G*′º), using a technique developed by Robert A. Alberty (5). Although Alberty proposed using several protonation states for some compounds, we simplified the technique by using only the unique protonation state stored in MetaCyc.

As of August 2015, a total of 12 386 compounds and 12 345 reactions included these Gibbs free energy values.

### Electron transfer pathways

**Figure 1.**
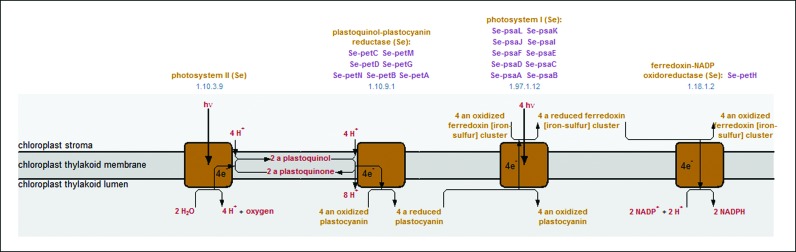
New electron transfer pathway diagrams enable connecting multiple reactions via any electron carrier. This type of diagram provides more information for electron transfer chains than does the typical metabolic pathway diagram, and clearly depicts the direction of the electron flow, the cell-compartment locations where the substrates are transformed, and the optional translocation of protons across membranes.

MetaCyc has contained simple electron transfer pathways for several years. This type of pathway utilizes a different display algorithm than the one used for standard metabolic pathways. The graphics for electron transfer pathways convey features such as the direction of the electron flow, the cell-compartment locations where the substrates are transformed, and the optional translocation of protons across membranes. However, those pathways were limited to only two reactions that had to be connected by a quinone-type electron carrier. We have enhanced this type of pathway to enable connecting an unlimited number of reactions, which can be connected to each other by any type of electron carrier. For example of such a pathway, see Figure 1.

### Quality assurance of MetaCyc data

Before each release of MetaCyc, we conduct a series of quality-assurance tests that expose errors in the data. A combination of automated and manual procedures is employed to find and correct problems, and to improve data accuracy and consistency. Although the full list of these procedures is too long to describe here, we would like to mention a few of them.

- **Constraint violations:** Programs check for violations of the constraints defined for many database attributes. For example, no object other than a compound or a protein should have a molecular weight value. We also check for cardinality violations (for example, a compound should have only a single value for its molecular weight).
- **Compound duplicates:** A program scans for potential duplicate compounds by comparing all possible pairs of chemical structures.
- **EC numbers:** A program checks that every EC number has at least one reaction associated with it, and that no reaction refers to a deleted or transferred EC number.
- **Inverse links:** These are links between two objects in the database that refer to each other. For example, if gene *x* refers to protein X as its product, then protein X should refer to gene *x* as the gene encoding it. The software ensures that all such links are reciprocal.
- **GO terms:** A program checks whether all GO terms are up-to-date, and enables the removal or replacement of all terms that have become obsolete.
- **Internal hyperlinks and HTML formatting:** A program checks that all internal hyperlinks point at valid targets, and that all HTML tags are balanced (e.g. every <i> tag is followed by an </i> tag).
- **Citations:** A program checks whether all PubMed citations in the database refer to valid PubMed IDs and that all such references have been properly imported.
- **Non-specific enzyme names:** A program compares all enzymatic activities in MetaCyc to a list of nonspecific names to ensure that the names describe a specific activity and to prevent meaningless matching between MetaCyc reactions and enzymes during the generation of a new PGDB (for example, the generic name ‘a kinase’ is not permitted in MetaCyc).

## EXPANSION OF BIOCYC

The BioCyc databases are organized into three tiers.

- Tier 1 PGDBs have received at least one year of manual curation. Although some Tier 1 PGDBs (e.g. MetaCyc and EcoCyc) have received decades of manual curation and are updated continuously, others are less well curated.
- Tier 2 PGDBs have received moderate (less than a year) amounts of review, and are usually not updated on an ongoing basis.
- Tier 3 PGDBs were created computationally and received no subsequent manual review or updating.

During the past two years, the number of BioCyc PGDBs increased from 2988 in version 17.1 to more than 5700 in version 19.0.

As of version 19.0, Tier 1 includes 7 PGDBs, Tier 2 includes 39 PGDBs, and Tier 3 includes more than 5700 PGDBs. Some Tier 2 PGDBs were provided by groups outside SRI. The database authors are identified on the database summary page (Analysis → Summary Statistics).

### Composition of Tier 3 genomes

Most of the genomes contained in BioCyc have been retrieved from the NCBI Reference Sequence Database (RefSeq) (6,7). Genomes included in Tier 3 are bacterial and archaeal genomes that have been labeled by RefSeq as Reference, Representative, or Complete Genomes.

Of the more than 5700 PGDBs found in BioCyc, >900 genomes are from the Human Microbiome Project (http://www.hmpdacc.org/catalog/grid.php?dataset=genomic&project_status=Complete).

## SOFTWARE AND WEBSITE ENHANCEMENTS

The following sections describe significant enhancements to Pathway Tools (the software that powers the BioCyc website) during the past 2 years.

### New layout for the gene/protein page

**Figure 2.**
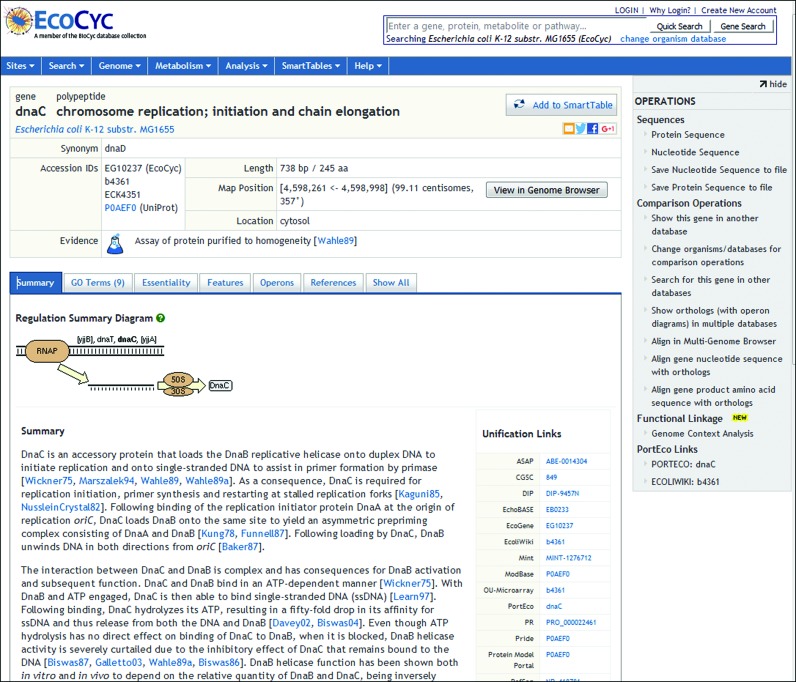
The new layout of the gene/protein page adds tabs, enabling the user to switch quickly among different data fields, including a summary, GO terms, essentiality data, protein features, operons, and references. A Show All tab places all the information on a single scrollable page.

The gene/protein page has been redesigned with a new tabbed layout to enhance the ability of users to find information, and to modernize its appearance (this feature will be released in mid-November 2015.) Some key information has been moved to the top of the page. Tabs enable the user to conveniently switch between different types of data without the need to scroll. The number of tabs is dynamic and depends on the available information. For example, ‘Essentiality’ and ‘GO terms’ tabs appear only if this type of data is available for the protein (see Figure 2).

### SmartTables enhancements

A SmartTable (previously called a Web Group) is a spreadsheet-like structure that can contain both PGDB objects and other data such as numbers or text. Like a spreadsheet, it is organized by rows and columns that the user can add to or delete. A typical SmartTable contains a set of PGDB objects in the first column (e.g. a set of genes generated by a search). The other columns contain properties of the object (e.g. the chromosomal position of each gene), or the result of a transformation (e.g. the reactions catalyzed by the gene products, or the corresponding genes from a different organism).

Since the last Nucleic Acids Research publication we have added several important capabilities to the SmartTables, including:

- **Creating** ‘**frozen**’ **copies:** This feature allows a user to publish a set of PGDB objects, such as a gene or metabolite list, within a publically readable, non-modifiable SmartTable. Because frozen SmartTables cannot be changed, they can be referred to in scientific publications.
- **Database links:** A new type of property column contains identifiers in an external database for SmartTable objects. For example, the user can select a column that contains BioCyc compounds and then add a new column that lists the ChEBI identifiers of these compounds.
- **Multiple databases:** A single SmartTable can hold objects from multiple BioCyc databases.
- **Sequence variant data analysis:** This new tool aids in analyzing how nucleotide-sequence variation can affect the protein product of a gene. The tool acts on SmartTables of replicon regions and associated sequence variants, which can be created easily via a file import operation. A new transformation ‘Sequence - nearest gene to DNA region’ adds additional columns to the SmartTable showing the nearest gene to each altered region, and the amino-acid change caused by each sequence substitution, insertion, or deletion.

### Improvements to pathway-based visualization of omics data

Pathway visualization of transcriptomics, metabolomics, and reaction-flux data facilitates the interpretation of such data by putting it within a biological context.

The following are among the enhancements to the Omics viewers made during the last two years.

#### Displaying omics data on individual pathway diagrams

**Figure 3.**
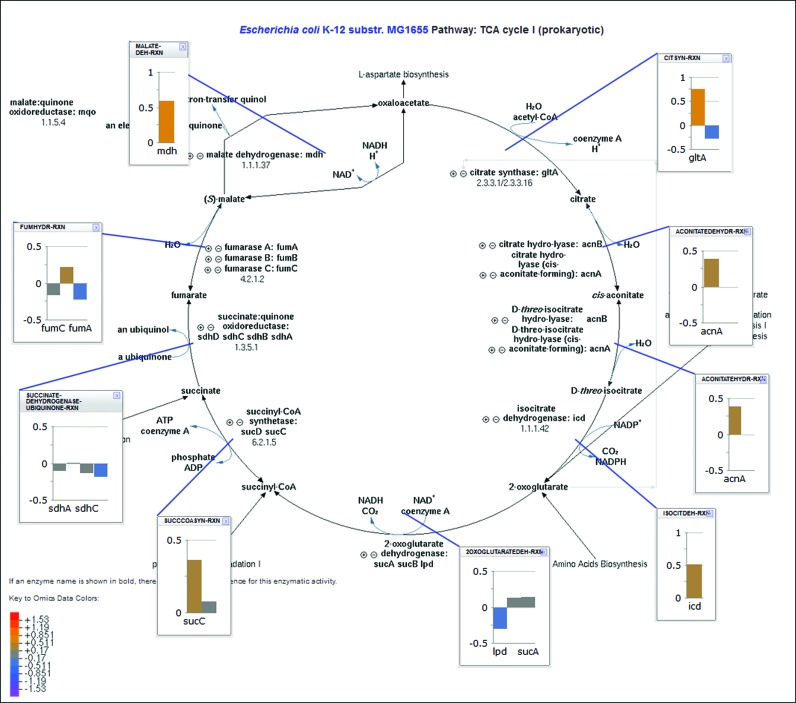
Omics datasets containing multiple time points can be presented in the form of omics popups on individual full-detail pathway diagrams. The user can select from several layouts and drag the popups to modify their location.

A new function enables the painting of omics data with multiple time points on individual pathway diagrams. The ‘Customize or Overlay Omics Data on Pathway Diagram’ operation, which is available from the right sidebar of every pathway page, lets the user upload an omics dataset with one or more time points. Datasets containing multiple time points are presented in the form of omics popups (see Figure 3), and the user can select from several layouts and drag the popups to modify their location.

#### More flexible omics-data file formats for omics viewers

The omics-data loading software has been enhanced to recognize a wider variety of gene and metabolite names and identifiers in the first column of the file. Genes and metabolites can now be specified using multiple alternative names and identifiers, including identifiers from other databases that are present in BioCyc, such as Chemical Entities of Biological Interest (ChEBI) (8) and Kyoto Encyclopedia of Genes and Genomes (KEGG) (9) IDs (for details see http://biocyc.org/PToolsWebsiteHowto.shtml#node_toc_node_sec_9.3.3).

#### Pathway collages

**Figure 4.**
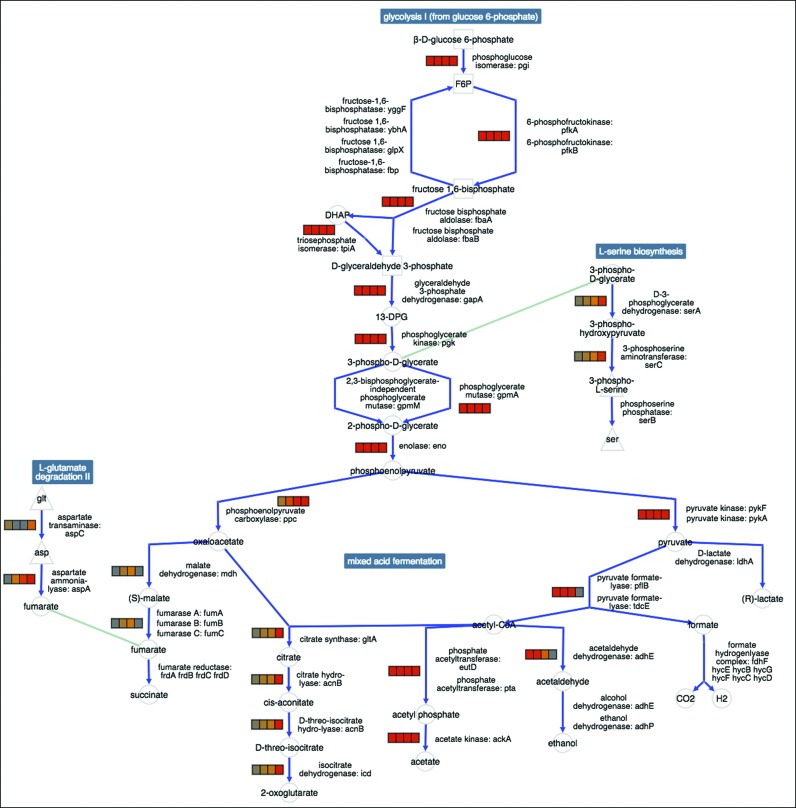
The new pathway collage diagram combines multiple pathway diagrams for a selected set of pathways. Users can edit pathway collages within their web browser, such as by adding additional pathways; zooming in or out; manually moving pathways or metabolite nodes; showing or hiding connections; highlighting objects of interest; and displaying omics data (transcriptomics data are shown in this image).

This new tool enables the user to create a diagram containing multiple pathway diagrams, called a pathway collage^1^. The individual pathways that are included in the collage can be specified in multiple ways, such as within a SmartTable. Once generated (from either the BioCyc website or a Pathway Tools desktop installation), a Pathway Collage can be manipulated via a web browser application that enables the user to customize the diagram in various ways, such as by zooming in or out, manually re-positioning pathways or metabolite, showing or hiding connections between metabolites, highlighting objects of interest, and displaying omics data. A Pathway Collage can be saved and later reloaded, or can be exported to a PNG image file to generate high quality images for use in publications and presentations (Figure 4).

### Search enhancements

#### Cross-organism search

This new tool, available from the Search menu, enables name-based searches across any specified set of organisms in BioCyc. For example, it is possible to search all cyanobacterial PGDBs for the text string ‘sulfolipid’. The user can restrict the search to a particular type of object, including proteins, enzymatic reactions, compounds, reactions, pathways, genes, and RNAs.

#### Search organism by phenotype

**Figure 5.**
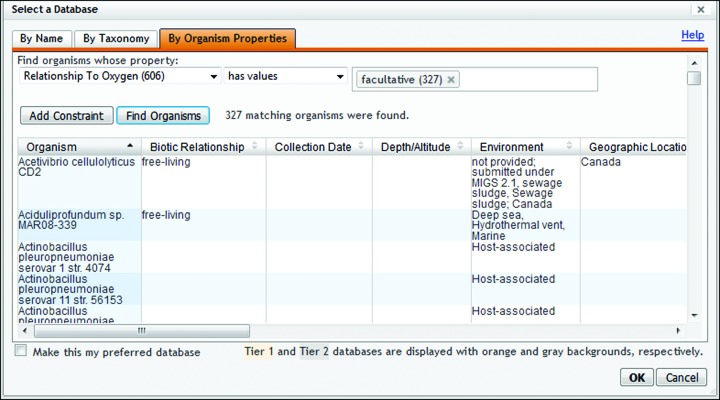
Adding MIGS [Minimal Information about a (Meta)Genome Sequence] data to PGDBs enables searching the organism list for organisms that match particular phenotypes. In this example, BioCyc was queried for PGDBs for organisms that are facultative in respect to oxygen, retrieving 327 matches.

Pathway Tools now supports the inclusion of MIGS [Minimal Information about a (Meta)Genome Sequence] data, which is a standard that defines the minimal essential information required to provide an accurate description of a genome and the organism or collection of organisms it was derived from (10). The MIGS data includes description of phenotypic data such as the temperature range and oxygen requirements or sensitivity of the organism. The new Organism Properties tab within the organism selector dialog enables users to search for BioCyc organisms according to these phenotypic properties (See Figure 5).

#### Sequence pattern search

This new tool, available from the Search menu, enables searching a genome for matches to short (<20) patterns of nucleotides or amino acids sequences. The tool scans a selected genome for matches to exact or degenerate sequences, returning a table with hyperlinks to all results.

### Other enhancements

#### Genome-context analysis

This new analysis tool generates Functionally Linked Gene Clusters (FunGCs) using genome-context methods, which are computational comparative-genomics methods that search for patterns of gene occurrence across 1800 reference genomes in BioCyc (11,12). These methods identify both pairwise functional linkages among genes, and larger clusters of genes that are likely to belong to a common pathway or process. Analysis of FunGCs can lead to discoveries of novel pathways and new insights on gene functions. FunGCs have only been calculated for a small number of Tier 1 and Tier 2 databases, but more will be added on a continual basis.

#### Metabolite translation service

This new tool, available from the Metabolism menu, translates metabolite names; identifiers; InChI strings; InChI keys; monoisotopic molecular weights; and molecular formulas, between metabolite databases. The metabolite data can be submitted to the metabolite translation service via a file or by pasting the data into a form (http://biocyc.org/metabolite-translation-service.shtml). The input is a series of lines, one per metabolite, where each line contains one or more identifying attributes, such as name; identifier from some metabolite database (including BioCyc); molecular formula; InChI key or string; or monoisotopic weight. If a unique match is found in BioCyc, the output will include a line listing the name for that metabolite, its BioCyc identifier, and the identifiers for that metabolite found in all other databases that BioCyc has data for. Ambiguous matches are also indicated.

#### Multiple sequence alignment tool

This new tool enables the user to align the sequence of a gene or its protein product with orthologs from a pre-defined group of organisms.

#### iPhone/iPad app expanded

**Figure 6.**
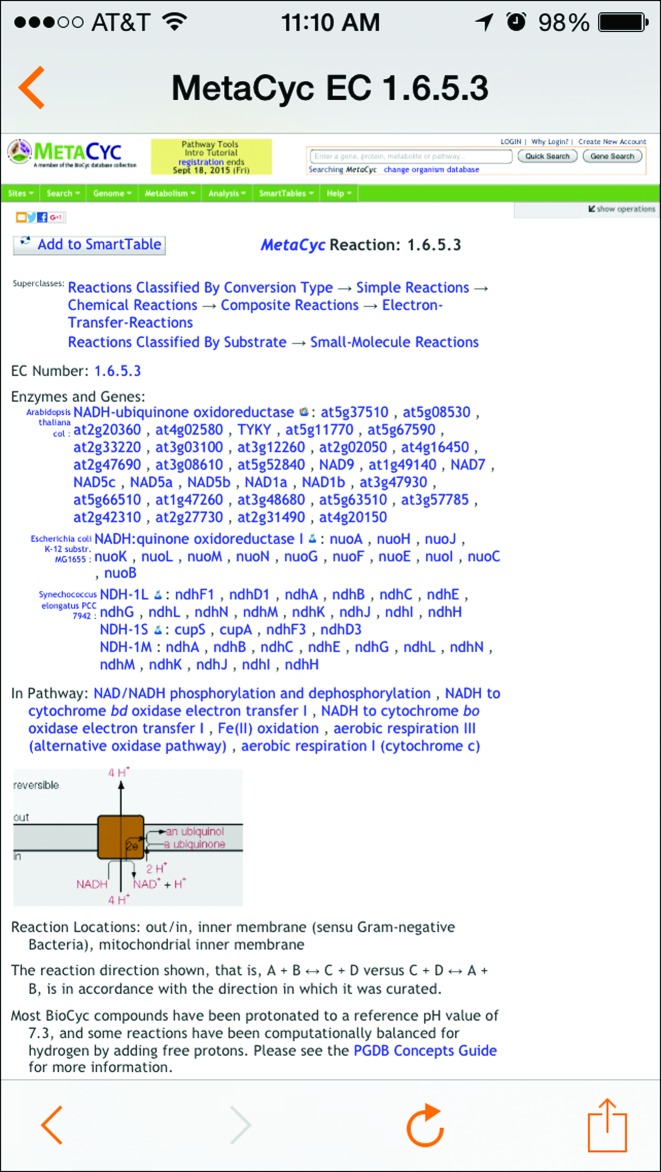
The new BioCyc app, which works on iOS devices, enables users to access any database in BioCyc, and any database in any other Pathway-Tools-based website that is running version 18.5 or later of Pathway Tools. The app lets users select a PGDB, query it for genes, and display related information, such as gene/protein data, catalyzed reactions, and metabolic pathways that the gene products participates in.

An EcoCyc app providing access only to the EcoCyc database and running only on an iPhone has been available for several years now. Starting in 2014, that app has been replaced with the new BioCyc app, which enables users to access any database in BioCyc, and any database in any other Pathway-Tools-based website that is running version 18.5 or later of Pathway Tools. The new app, which also works on the iPad, is available at the Apple iTunes store (Figure 6).

#### PythonCyc

A new Python API is available for Pathway Tools. It supports programmatic querying and updating of PGDBs via Python. More than 160 functions of Pathway Tools can be called from PythonCyc. Like PerlCyc and JavaCyc, PythonCyc can perform basic operations for querying and modifying a PGDB. However, PythonCyc also contains an advanced set of operations to represent frame objects as Python objects. PythonCyc can also be accessed remotely, that is, a Python interpreter can execute on a different computer than the computer executing Pathway Tools. Installation instructions, a tutorial, and the complete list of Pathway Tools’ functions callable from Python are available at http://bioinformatics.ai.sri.com/ptools/pythoncyc.html.

#### Web services enhancements

New web services enable retrieving BioCyc metabolite IDs in bulk by either monoisotopic molecular weight or chemical formula. In addition, several new web services provide access to SmartTables, allowing creation, deletion, and modification of SmartTables and their rows and columns. See http://biocyc.org/web-services.shtml for the full description of available web services.

### MetaFlux metabolic modeling enhancements

MetaFlux, the Flux Balance Analysis (FBA) module of Pathway Tools, has been enhanced to enable creating models of organism communities in which several organisms can compete and cooperate in a user-specified growth environment. MetaFlux achieves this by applying dynamic FBA (dFBA) (first described by Varma *et al*. (13)) to a community of organisms by a technique similar to that used by Harcombe *et al*. (14) and Zhuang *et al*. (15), which they termed dynamic multi-species metabolic modeling (DMMM). A similar technique has also been implemented by the COBRA (COnstraints-Based Reconstruction and Analysis) group (16).

dFBA enables executing community models over a given time period. The initial conditions of a community model specify starting biomasses for each organism in the simulation; the nutrients supplied to the organisms; the death rate of the organisms; the number of time steps to simulate; and the real time for each step. For each organism in the community, an FBA model is supplied. Executing the community model consists of solving the individual FBA models at each step and recording each organism's nutrient uptake and secretion rate.

A community model may optionally be executed within a two-dimensional spatial grid, such as to model a cross-section through the human gut. Each grid square can be specified to contain different organism abundances and different nutrient sets. New organisms and nutrients can be introduced during execution of either a grid model or a non-grid model. Diffusion of metabolites and organisms are simulated in grid models, based on their diffusion coefficients.

The resulting simulation of a community can be plotted graphically, including plotting the organisms’ total biomasses over time, and the concentrations of nutrients and secreted compounds. A more detailed description of the implementation of dFBA in MetaFlux has been published recently (17).

## ENHANCEMENTS TO THE DESKTOP VERSION OF PATHWAY TOOLS

The following enhancements only apply to the desktop version of the Pathway Tools software.

### Sequence editor

This new tool, available from the Chromosome menu, enables the user to manually update a replicon nucleotide sequence. This tool is intended for making relatively small local changes. A different tool is available for making global sequence changes.

### SBML file importer

A new tool, available under the File menu, enables importing an SBML file into a new or existing PGDB. The tool creates all metabolites and reactions defined in the SBML file in the PGDB, and attempts to map those metabolites and reactions to the metabolites and reactions contained in MetaCyc. In addition, the SBML exporter was augmented to include many more database links to external database resources, in a format that is commonly used by the COBRA Toolbox.

### Ability to import phenotype microarray data into a PGDB

We have added the capability to import phenotype microarray data into a PGDB from a discretized OPM YAML file, which is generated by OPM (an R package designed to analyze multidimensional OmniLog® phenotype microarray data) (18). Once imported, the data can be displayed using existing Pathway Tools functionality, and compared to metabolic model results.

### Editor for organism phenotype information

The PGDB Info Editor has been extended to support entry and updating of organism phenotype data, such as the temperature range and aerobicity of the organism via the MIGS [Minimal Information about a (Meta)Genome Sequence] standard.

## HOW TO LEARN MORE ABOUT METACYC AND BIOCYC

The BioCyc.org and MetaCyc.org websites provide several informational resources, including an online BioCyc guided tour (http://biocyc.org/samples.shtml); a guide to the BioCyc database collection (http://biocyc.org/BioCycUserGuide.shtml); a guide for MetaCyc (http://www.metacyc.org/MetaCycUserGuide.shtml); a guide for EcoCyc (http://biocyc.org/ecocyc/EcoCycUserGuide.shtml); a guide to the concepts and science behind Pathway/Genome Databases (http://biocyc.org/PGDBConceptsGuide.shtml); and instructional webinar videos that describe the usage of BioCyc and Pathway Tools (http://biocyc.org/webinar.shtml). We routinely host workshops and tutorials (on site and at conferences) that provide training and in-depth discussion of our software for both beginning and advanced users. To stay informed about the most recent changes and enhancements to our software, please join the BioCyc mailing list at http://biocyc.org/subscribe.shtml. A list of our publications is available online at http://biocyc.org/publications.shtml.

## DATABASE AVAILABILITY

The MetaCyc and BioCyc databases are freely and openly available to all. See http://biocyc.org/download.shtml for download information. New versions of the downloadable data files and of the BioCyc and MetaCyc websites are released three times per year. Access to the website is free; users are required to register for a free account after viewing more than 30 pages in a given month.

## Footnotes

1 This tool will be released in November 2015.
